# Supplementary material for: Deciphering the critical role of interstitial volume in glassy sulfide superionic conductors
Source: Nat Commun. 2024 Mar 22;15:2552. doi: 10.1038/s41467-024-46798-4 (PMC10957893; doi:10.1038/s41467-024-46798-4)
Supplement: Supplementary file 1 — Supplementary Information [file 41467_2024_46798_MOESM1_ESM.pdf]

Supplementary Information

Deciphering the critical role of interstitial volume in glassy sulfide superionic conductors

Han Su[1,2], Yu Zhong[1,*], Changhong Wang[2,3,*], Yu Liu[1,2], Yang Hu[2], Jingru Li[1], Minkang Wang[1], Longan Jiao[4], Ningning Zhou[4], Bing Xiao[4], Xiuli Wang[1], Xueliang Sun[2,*], Jiangping Tu[1,*]

* Corresponding author

Email: yu_zhong@zju.edu.cn; cwang@eitech.edu.cn; xsun9@uwo.ca; tujp@zju.edu.cn;

[1] State Key Laboratory of Silicon Materials, Key Laboratory of Advanced Materials and Applications for Batteries of Zhejiang Province, School of Materials Science and Engineering, Zhejiang University, Hangzhou 310027, China.

[2] Department of Mechanical and Materials Engineering, University of Western Ontario1151 Richmond St., London, Ontario N6A 3K7, Canada

[3] Eastern Institute for Advanced Study, Eastern Institute of Technology, Ningbo, Zhejiang 315200, P.R. China.

[4] Carl Zeiss (Shanghai) Co., Ltd., 60 Mei Yue Road, Pilot Free Trade Zone, Shanghai, 200131, P. R. China

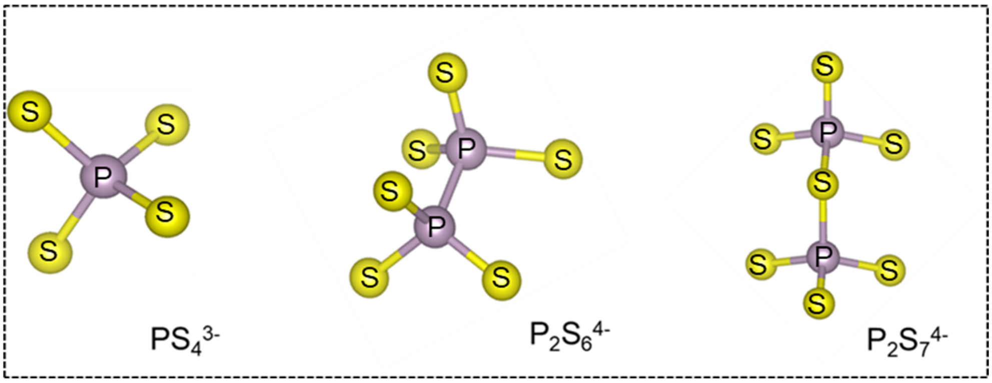

Fig. S1 Structures of typical anion clusters in $75Li_2S$-$25P_2S_5$ glass.

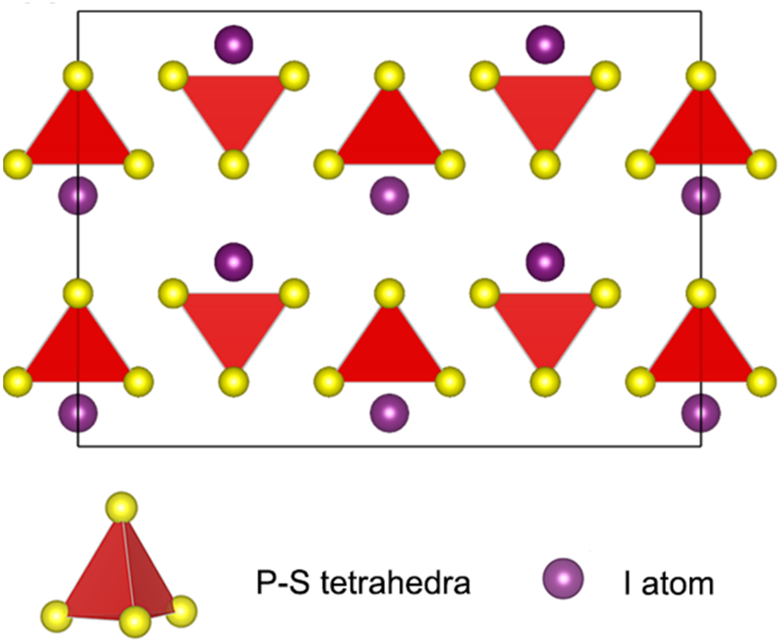

Fig. S2 The crystal structure of Li_4PS_4I.

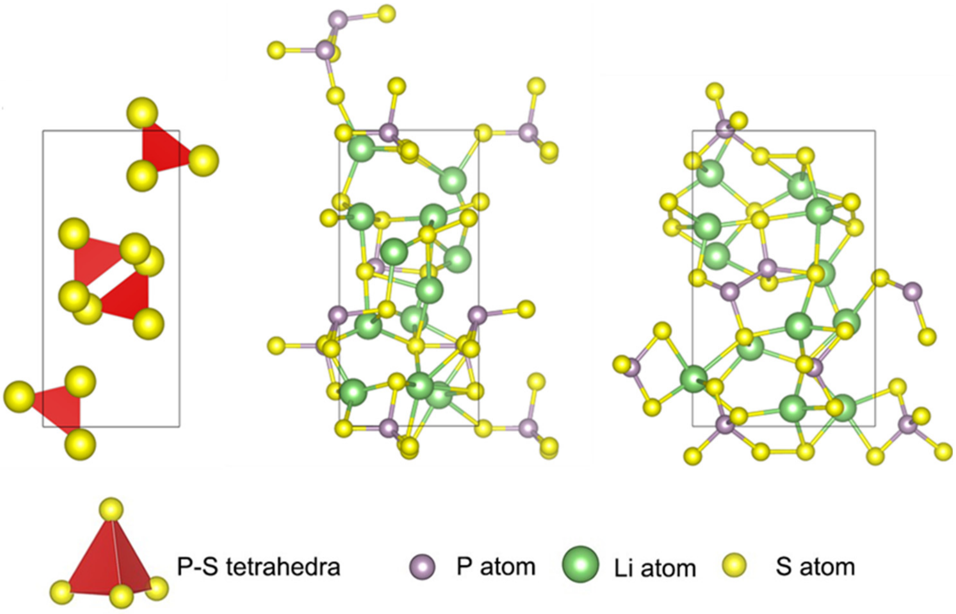

Fig. S3 Melt-quenching simulation results of β-Li_3PS_4. The structure of anion clusters in the β-Li_3PS_4 crystal (**left**). A snapshot of 75Li_2S-25P_2S_5 glass exhibiting the existence of $P_2S_7^{4-}$ after equilibrium (**middle**). A snapshot of 75Li_2S-25P_2S_5 glass exhibiting the existence of $P_2S_6^{4-}$ after equilibrium (**right**).

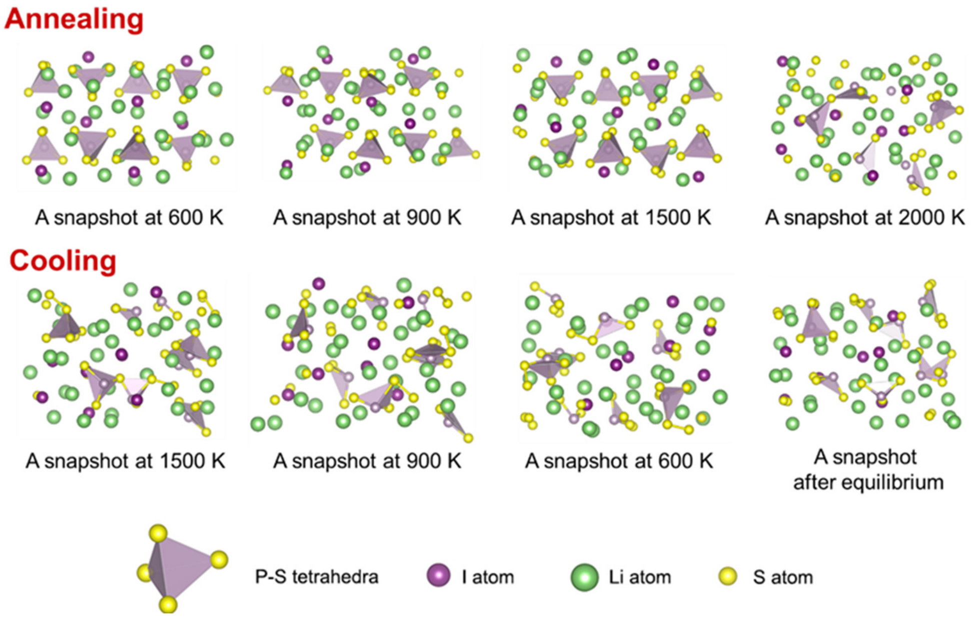

Fig. S4 Snapshots of Li_4PS_4I at different temperatures during the annealing-cooling process.

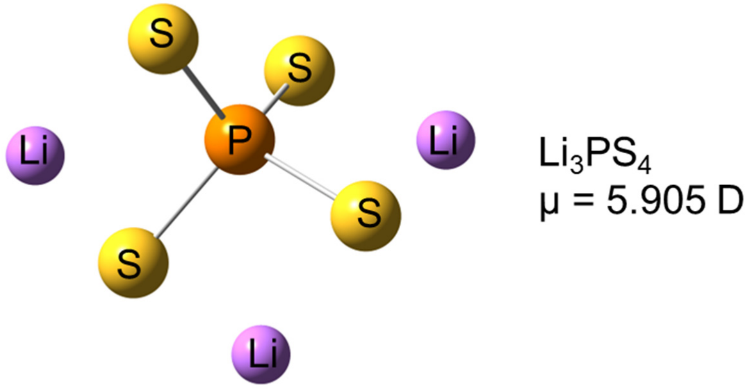

Fig. S5 The dipole moment of the Li_3PS_4 molecule.

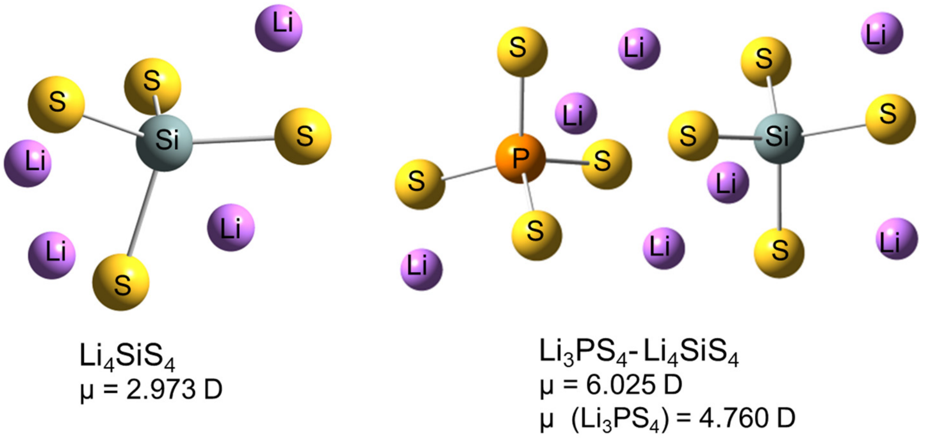

Fig. S6 The dipole moment of the Li_4SiS_4 molecule, Li_3PS_4-Li_4SiS_4 complex and Li_3PS_4 molecule after complexing with Li_4SiS_4.

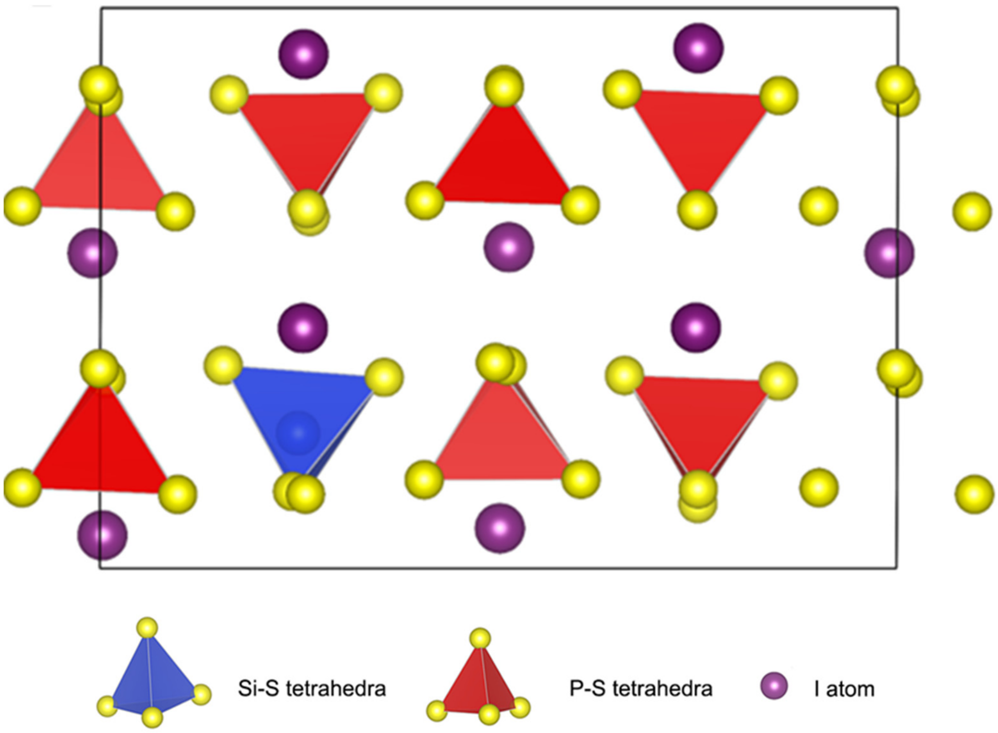

Fig. S7 The crystal structure of $Li_{4.125}Si_{0.125}P_{0.875}S_4I$.

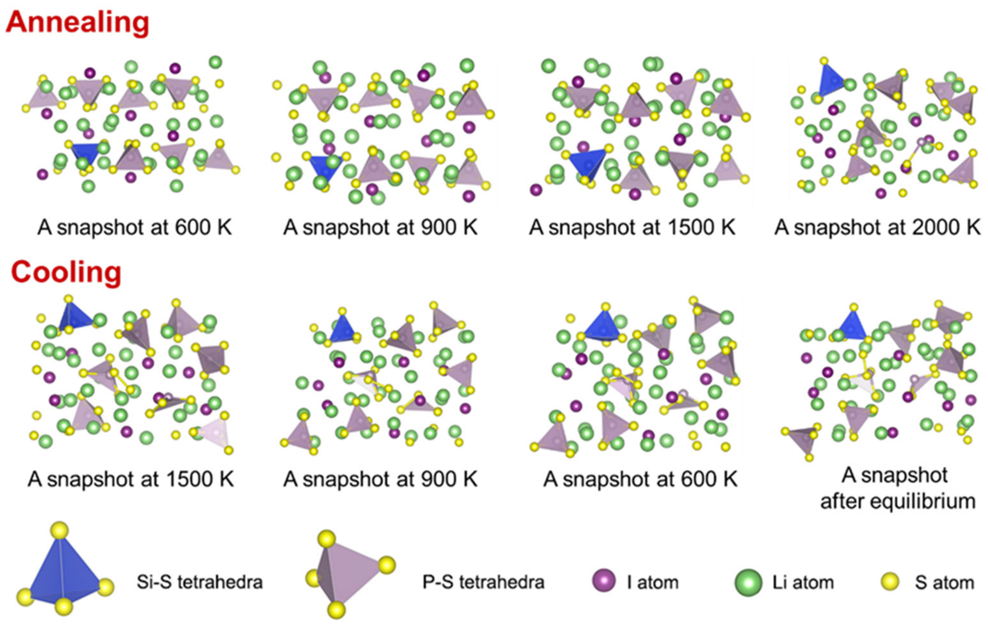

Fig. S8 Snapshots of $Li_{4.125}Si_{0.125}P_{0.875}S_4I$ at different temperatures during the annealing-cooling process.

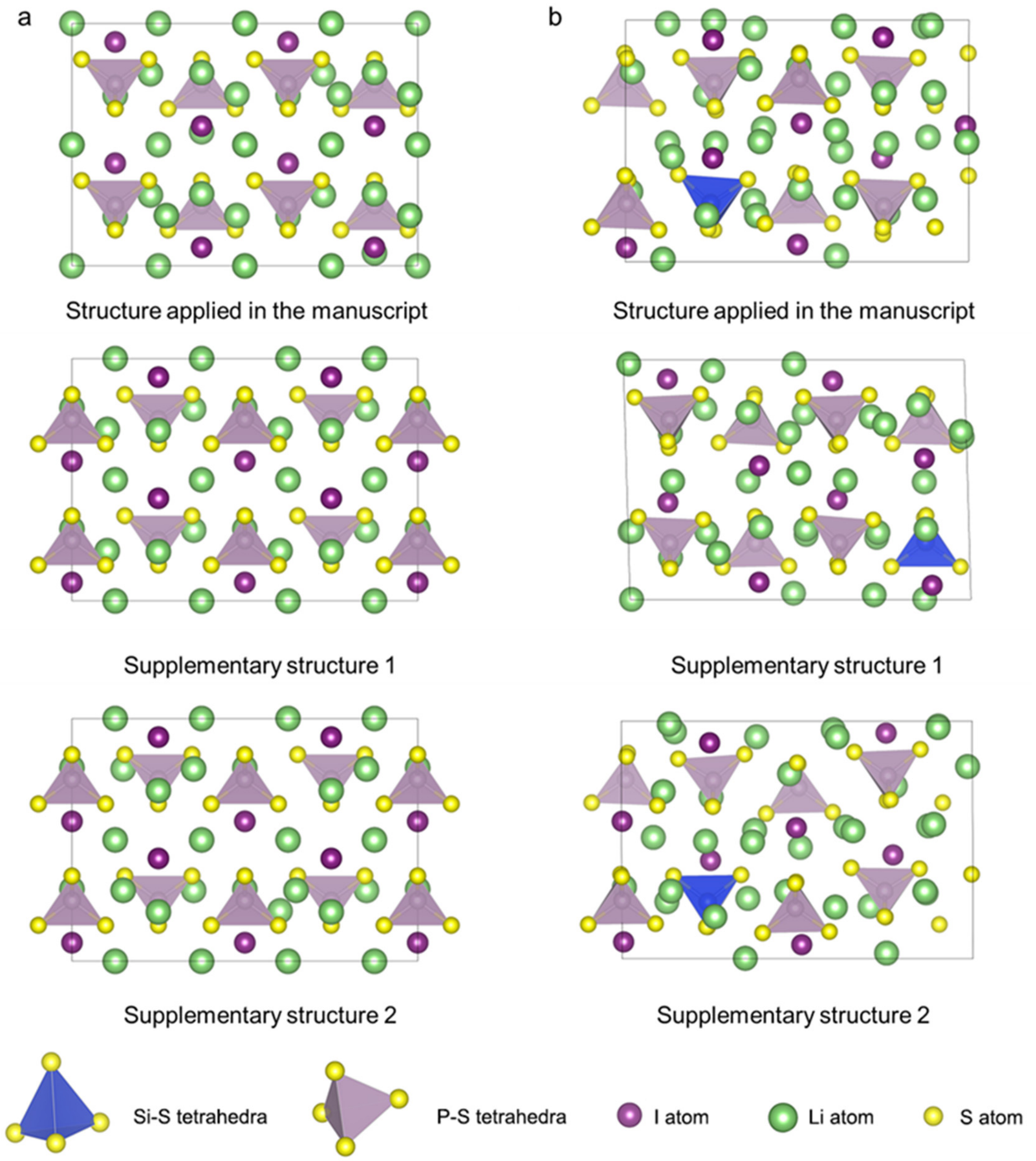

Fig. S9 The crystal structures of Li_4PS_4I and $Li_{4.125}Si_{0.125}P_{0.875}S_4I$ applied in the manuscript and employed for validation. **a** The crystal structure of Li_4PS_4I applied in the manuscript and other two additional structure employed for supplementary validation. **b** The crystal structure of $Li_{4.125}Si_{0.125}P_{0.875}S_4I$ applied in the manuscript and other two additional structure employed for supplementary validation.

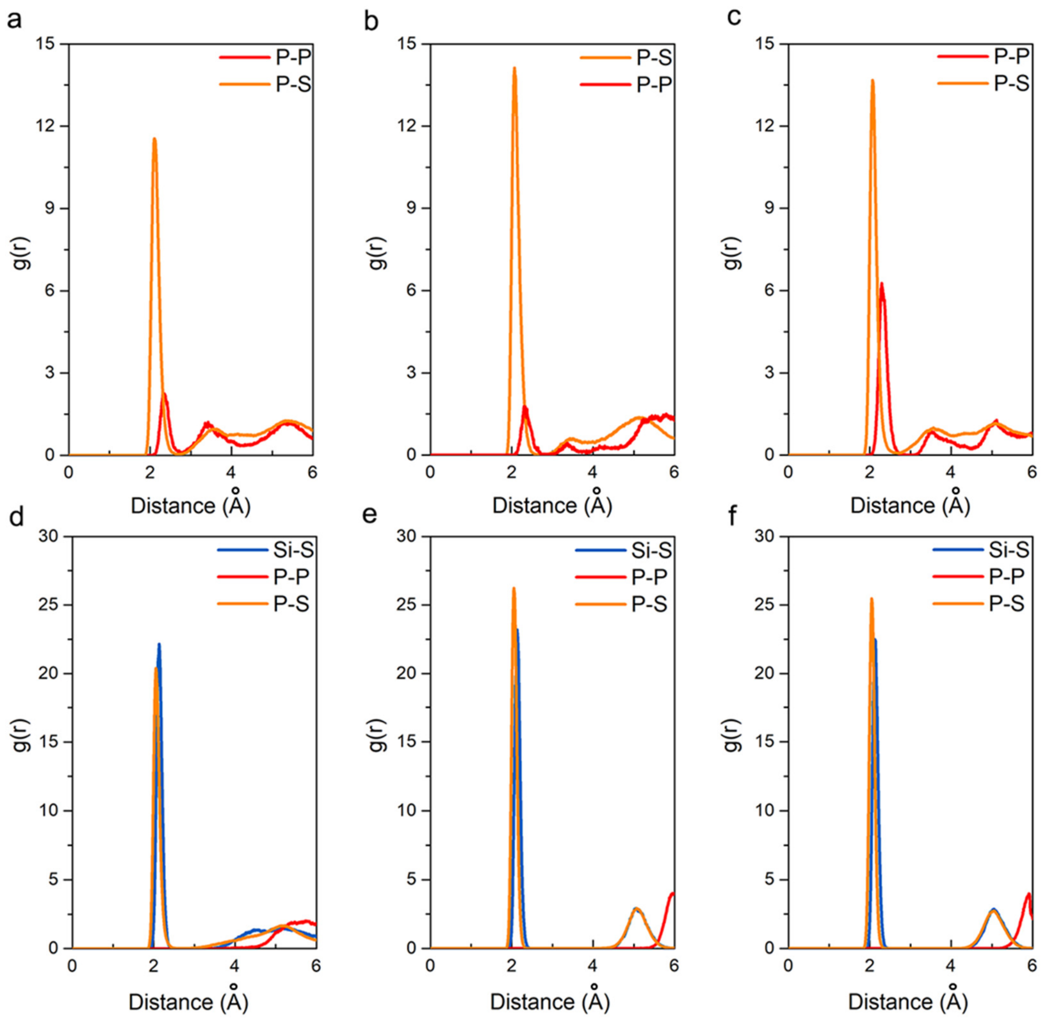

Fig. S10 The comparison of g(r) profiles in simulated glassy electrolytes with different initial structures. **a** The g(r) profiles of P-P and P-S pairs in the Li_4PS_4I glass with the structure applied in the manuscript. **b** The g(r) profiles of P-P and P-S pairs in the Li_4PS_4I glass with the Supplementary structure 1. **c** The g(r) profiles of P-P and P-S pairs in the Li_4PS_4I glass with the Supplementary structure 2. **d** The g(r) profiles of Si-S, P-P and P-S pairs in the $Li_{4.125}Si_{0.125}P_{0.875}S_4I$ glass with the structure applied in the manuscript. **e** The g(r) profiles of Si-S, P-P and P-S pairs in the $Li_{4.125}Si_{0.125}P_{0.875}S_4I$ glass with the Supplementary structure 1. **f** The g(r) profiles of Si-S, P-P and P-S pairs in the $Li_{4.125}Si_{0.125}P_{0.875}S_4I$ glass with the Supplementary structure 2.

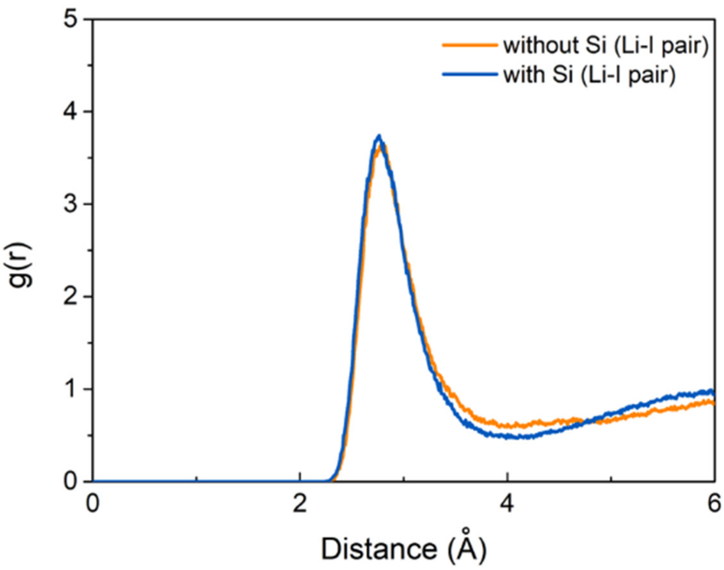

Fig. S11 The g(r) profiles of the Li-I pair in the Li_4PS_4I glass and the Si-doped $Li_{4.125}Si_{0.125}P_{0.875}S_4I$ glass.

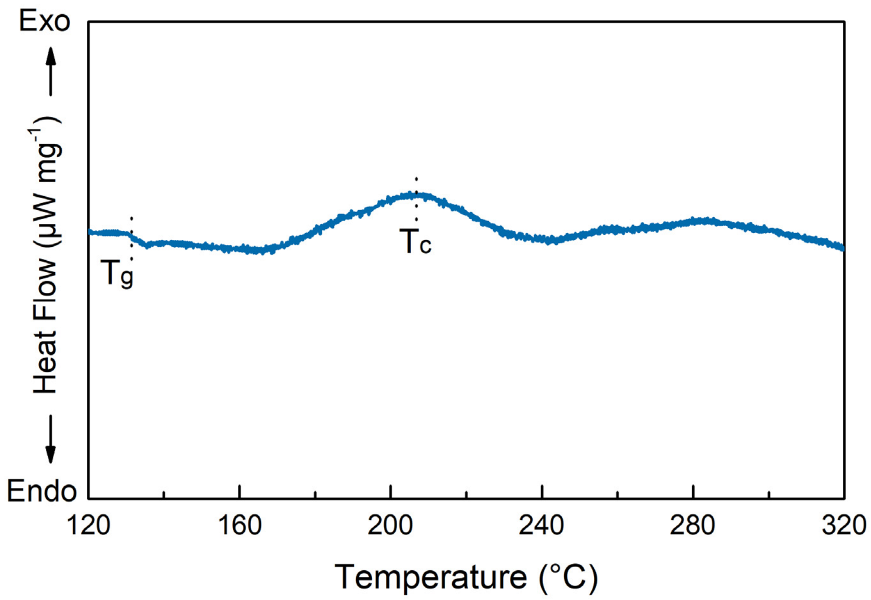

Fig. S12 The differential scanning calorimetry curve of the P:Si = 6 electrolyte in N_2 flow.

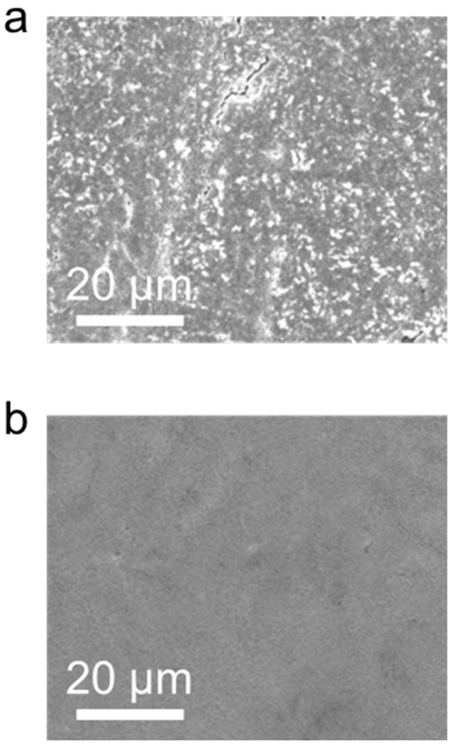

Fig. S13 The surface morphology of the cold-pressed and hot-pressed P:Si = 6 electrolytes. **a** The surface morphology of the cold-pressed P:Si = 6 electrolyte. **b** The surface morphology of the highly-densified hot-pressed P:Si = 6 electrolyte.

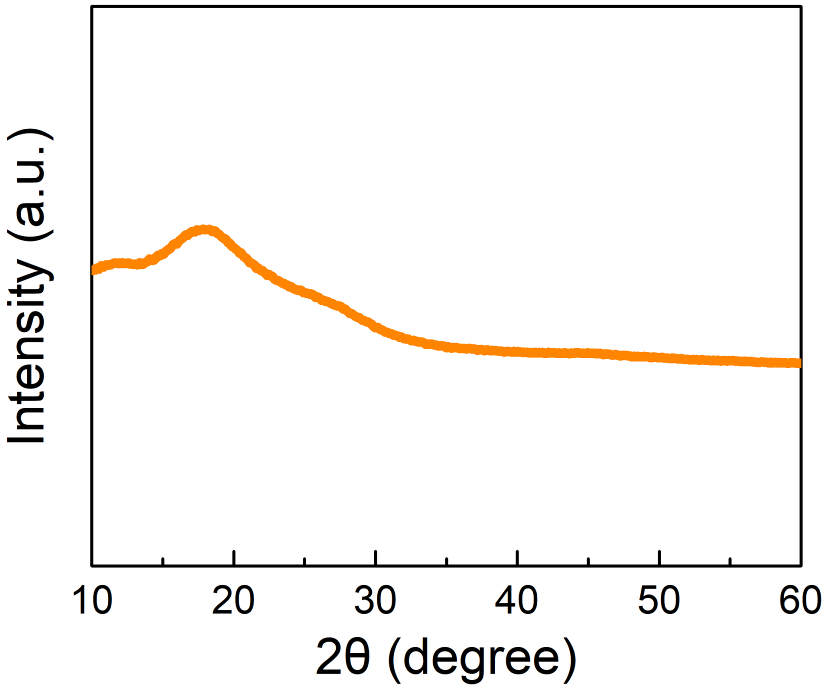

Fig. S14 The XRD spectrum of the hot-pressed P:Si = 6 electrolyte.

Table S1 The calculation of relative density of the P:Si = 6 electrolyte.

Composition	Theoretical Density / (g cm^{-3})	Weight / (mg)	Thickness / (mm)	Real Density / (g cm^{-3})	Relative Density
P:Si = 6	2.483	158.6	0.869	2.325	0.936

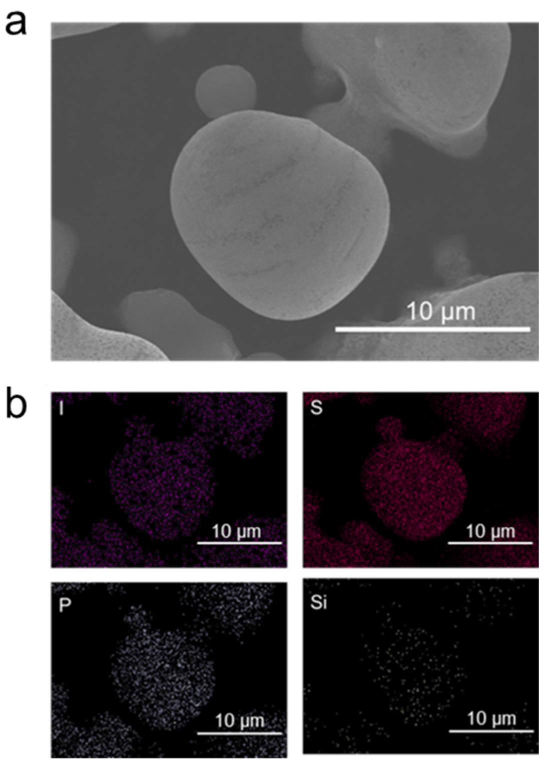

Fig. S15 The morphology and elemental distribution of the P:Si = 6 particle. **a** The particle morphology of the P:Si = 6 particle and **b** the corresponding EDS mapping results.

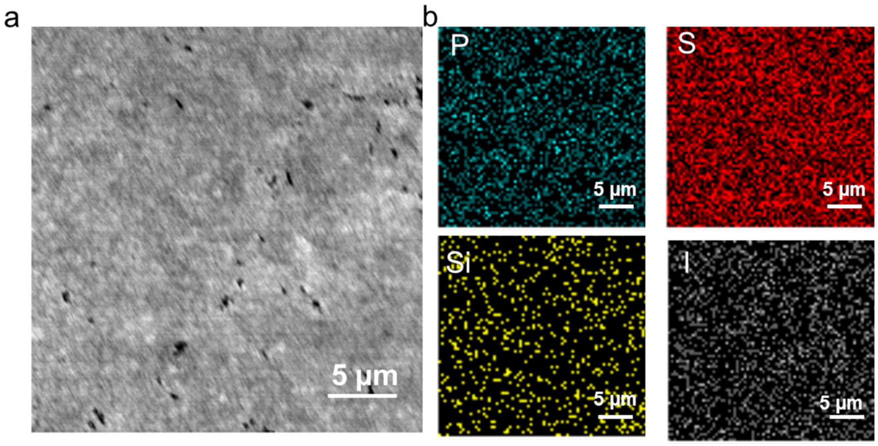

Fig. S16 The morphology and elemental distribution of the P:Si = 6 pellet. **a** The surface morphology of the P:Si = 6 pellet and **b** the corresponding EDS mapping results.

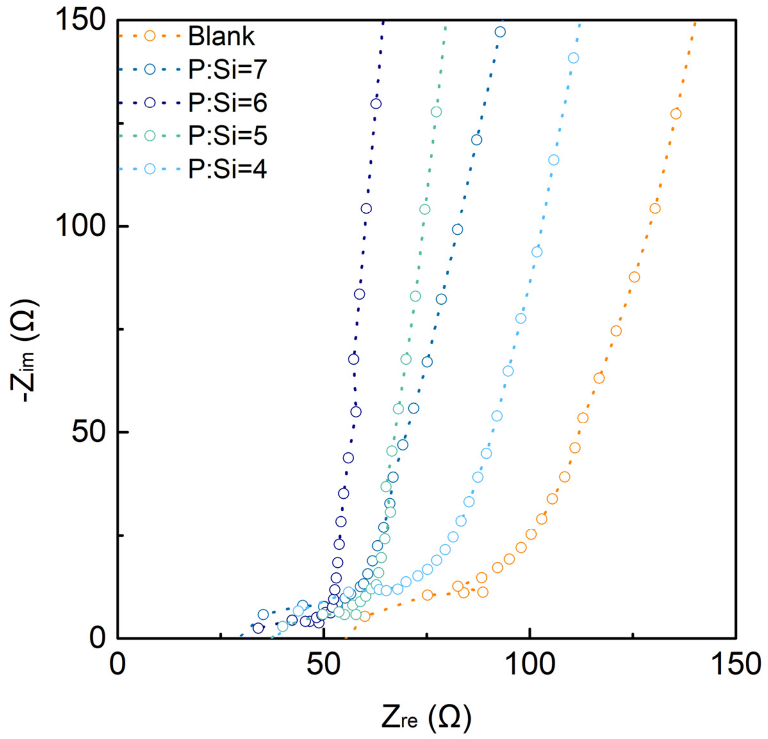

Fig.S17 Nyquist plots of electrolytes with different P/Si ratios at 298 K.

Table S2 The summary of the ionic conductivity and density of electrolytes with different P/Si ratios.

Composition	Blank	P:Si = 7	P:Si = 6	P:Si = 5	P:Si = 4
Resistance / (ohms)	101.20	58.68	50.17	61.83	73.18
Thickness / (mm)	0.885	0.872	0.869	0.874	0.879
Ionic conductivity / (mS cm^{-1})	1.11	1.90	2.21	1.80	1.53

Table S3 The summary of the reported ionic conductivity of glass sulfide electrolytes.

Glass Sulfide-based SE	Ionic Conductivity / (mS cm^{-1})	Ref.
Blank(60(0.75Li_2S-0.25P_2S_5)-40LiI)	1.11	*This Work
P : Si = 7	1.90	*This Work
P : Si = 6	2.21	*This Work
P : Si = 5	1.80	*This Work
P : Si = 4	1.53	*This Work
75Li_2S·25P_2S_5	0.50	1
70(0.75Li_2S-0.25P_2S_5)-30LiI	1.78	2
$Li_{0.84}B_{0.40}Si_{0.2}O_{0.4}S_{0.84}I_{0.36}$	2.10	3
67(0.75Li_2S·0.25P_2S_5)-33$LiBH_4$	1.60	4
0.26B_2S_3-0.30Li_2S-0.44LiI	1.70	5
0.28SiS_2-0.42Li_2S-0.30LiI	1.80	6
77.5Li_2S·22.25P_2S_5·0.25P_2O_5	1.11	7
30LiI·70(0.07Li_2O·0.68Li_2S·0.25P_2S_5)	1.30	8
0.07Li_2O·0.68Li_2S·0.25P_2S_5	0.25	8
80(0.7Li_2S·0.3P_2S_5)·20LiBr	0.34	9
75Li_2S·21P_2S_5·4P_2O_5	0.10	10

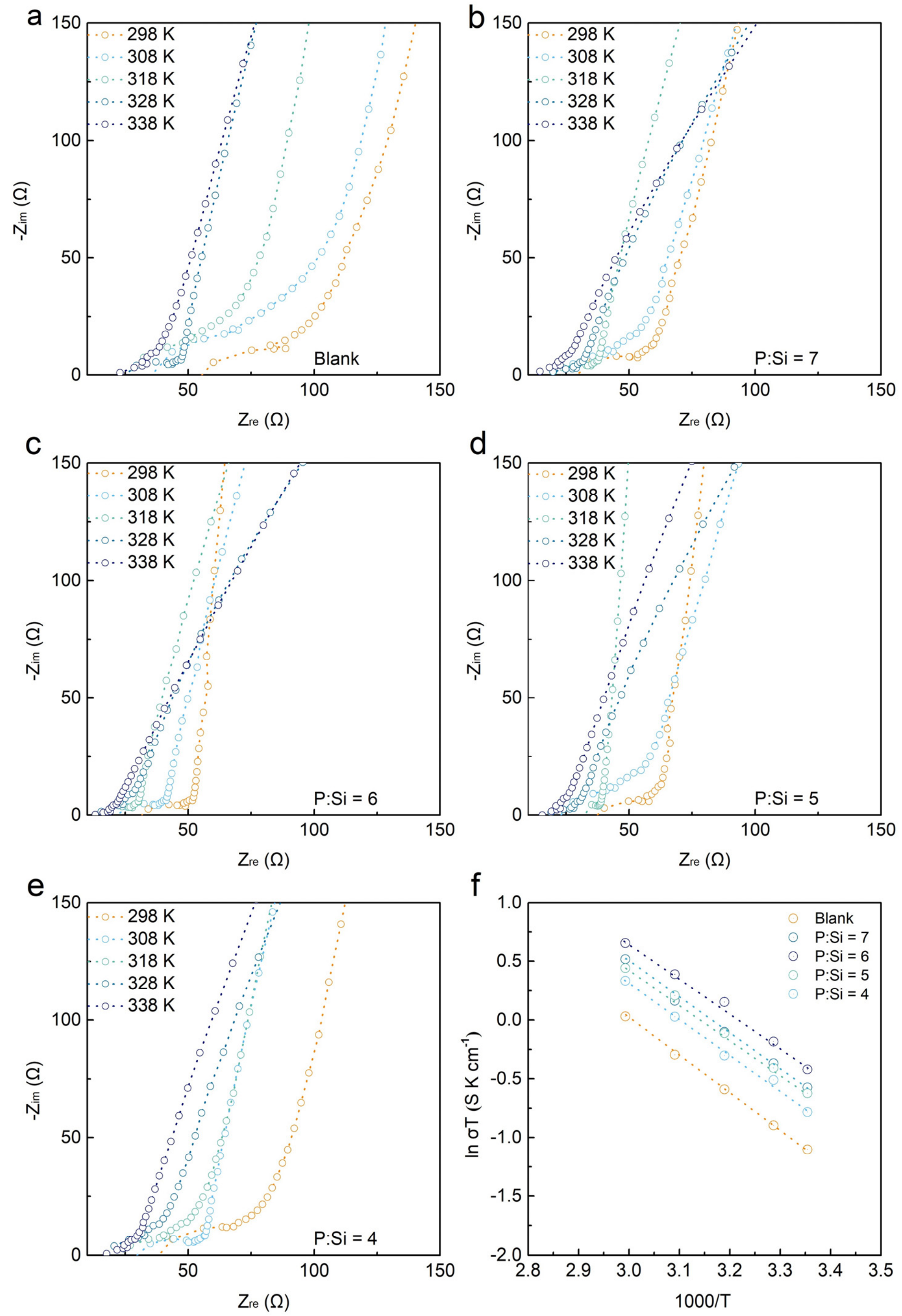

Fig. S18 The temperature-dependent Nyquist plots of synthesized electrolytes and the derived Arrhenius plot. **a** The temperature-dependent Nyquist plots of the blank electrolyte. **b** The temperature-dependent Nyquist plots of the P:Si = 7 electrolyte. **c** The temperature-dependent Nyquist plots of the P:Si = 6 electrolyte. **d** The temperature-dependent Nyquist plots of the P:Si = 5 electrolyte. **e** The temperature-dependent Nyquist plots of the P:Si = 4 electrolyte. **f** Arrhenius plots of electrolytes with different P/Si ratios.

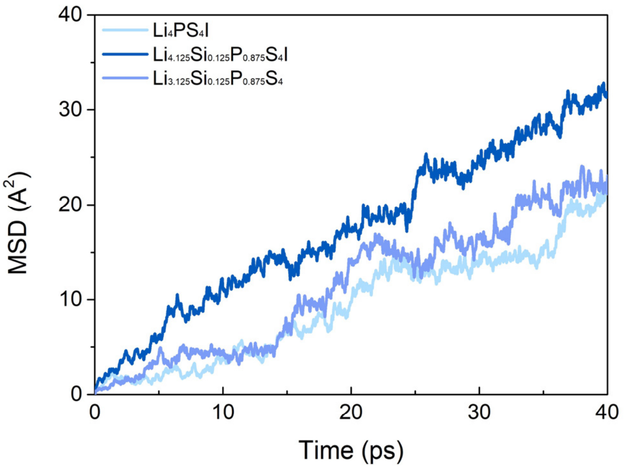

Fig. S19 The lithium MSD plots for Li_4PS_4I, $Li_{4.125}Si_{0.125}P_{0.875}S_4I$ and $Li_{3.125}Si_{0.125}P_{0.875}S_4$ at 600 K from AIMD simulations.

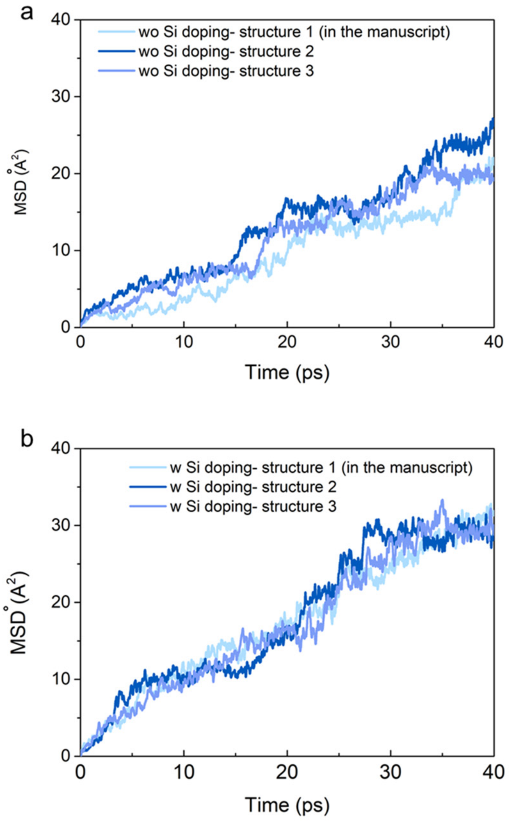

Fig. S20 The lithium MSD plots for three structures of Li_4PS_4I and $Li_{4.125}Si_{0.125}P_{0.875}S_4I$ glass. **a** The lithium MSD plots for three structures of Li_4PS_4I glass at 600 K from AIMD simulations. **b** The lithium MSD plots for three structures of $Li_{4.125}Si_{0.125}P_{0.875}S_4I$ glass at 600 K from AIMD simulations.

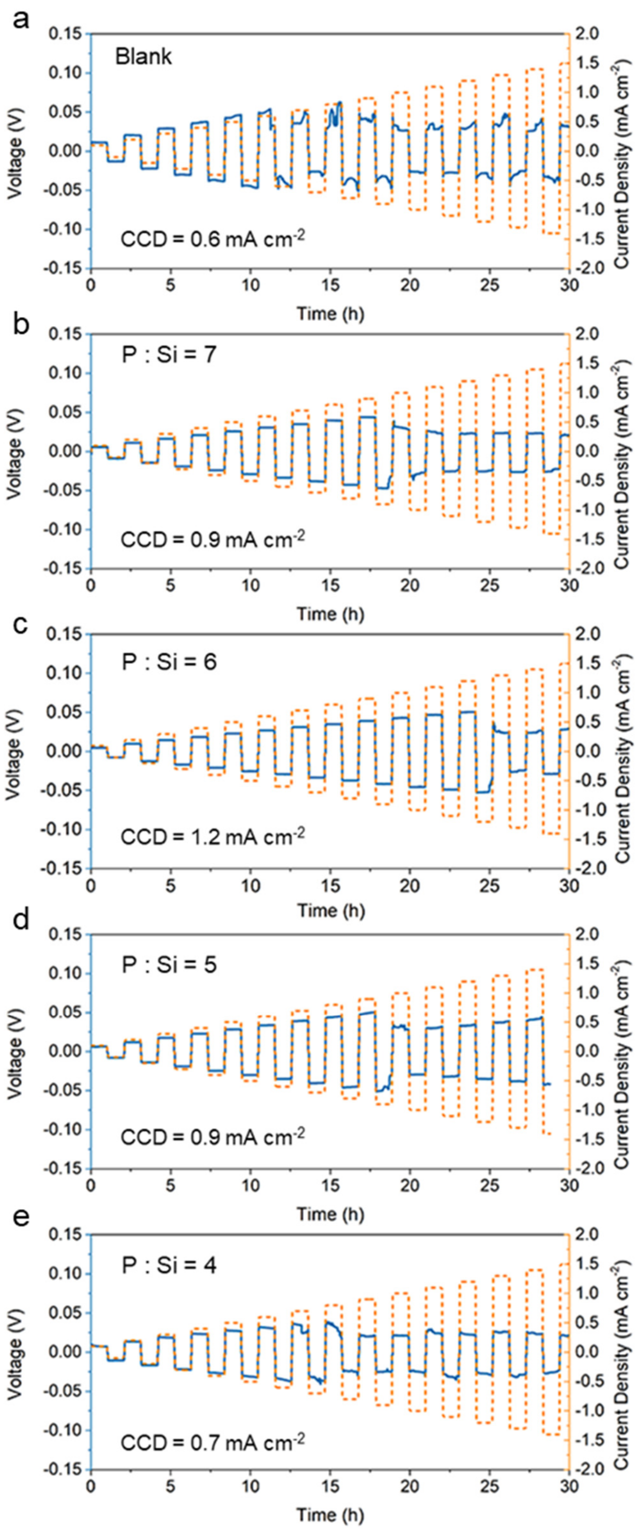

Fig. S21 CCD tests of synthesized electrolytes. **a-e** The voltage profiles of blank (**a**), P:Si = 7 (**b**), P:Si = 6 (**c**), P:Si = 5 (**d**) and P:Si = 4 (**e**) electrolytes during the CCD test. The step size of current density was set to be 0.1 mA cm^{-2}. And the charging/discharing time was set to be 1 h.

Table S4 The summarization of the reported CCD values for glass sulfide electrolytes.

Glass Sulfide-based SE	CCD / (mA cm^{-2})	Capacity / (mAh cm^{-2})	Ref.
P : Si = 6	1.2	1.2	*This Work
P : Si = 6	4.0	0.1	*This Work
$75Li_2S$-$25P_2S_5$	0.4	0.4	2
$70(0.75Li_2S$-$0.25P_2S_5)$-30LiI	1.0	1.0	1
$0.67Li_2S$- $0.33(0.2P_2O_5$-$0.8SiS_2)$	0.3	0.3	11
$60Li_2S$-$28P_2S_5$-$12SiS_2$ (Wafer)	1.8 (3MPa)	0.9	12
$60Li_2S$-$28P_2S_5$-$12SiS_2$ (Wafer)	0.6(1MPa)	0.3	12

Table S5 The summarization of the reported Li symmetric cell performances for glass sulfide electrolytes.

Glass Sulfide-based SE	Current Density / (mA cm^{-2})	Capacity / (mAh cm^{-2})	Cycling Time / (h)	Ref.
P : Si = 6	0.1	0.5	8000	*This Work
P : Si = 6	0.2	0.2	2000	*This Work
P : Si = 6	1.0	0.1	1500	*This Work
$70(0.75Li_2S$-$0.25P_2S_5)$-30LiI	0.3	0.3	200	2
$75Li_2S$-$25P_2S_5$	0.3	0.3	18	13
$Li_{0.84}B_{0.40}Si_{0.2}O_{0.4}S_{0.84}I_{0.36}$	0.1	0.1	110	3
$77.5Li_2S$-$22.25P_2S_5$-$0.25P_2O_5$	0.1	0.1	120	7

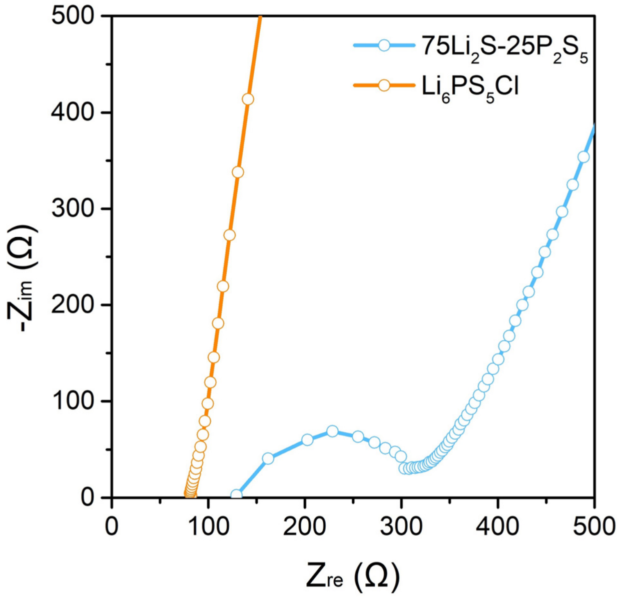

Fig. S22 Nyquist plots of Li_6PS_5Cl and $75Li_2S$-$25P_2S_5$ electrolytes at 298 K.

Table S6 The summary of relative density of Li_6PS_5Cl and $75Li_2S$-$25P_2S_5$ electrolytes.

Composition	Theoretical Density / (g cm^{-3})	Weight/ (mg)	Thickness / (mm)	Real Density / (g cm^{-3})	Relative Density
Li_6PS_5Cl	1.864	159.1	1.284	1.578	0.848
$75Li_2S$-$25P_2S_5$	1.865	157.3	1.181	1.697	0.910

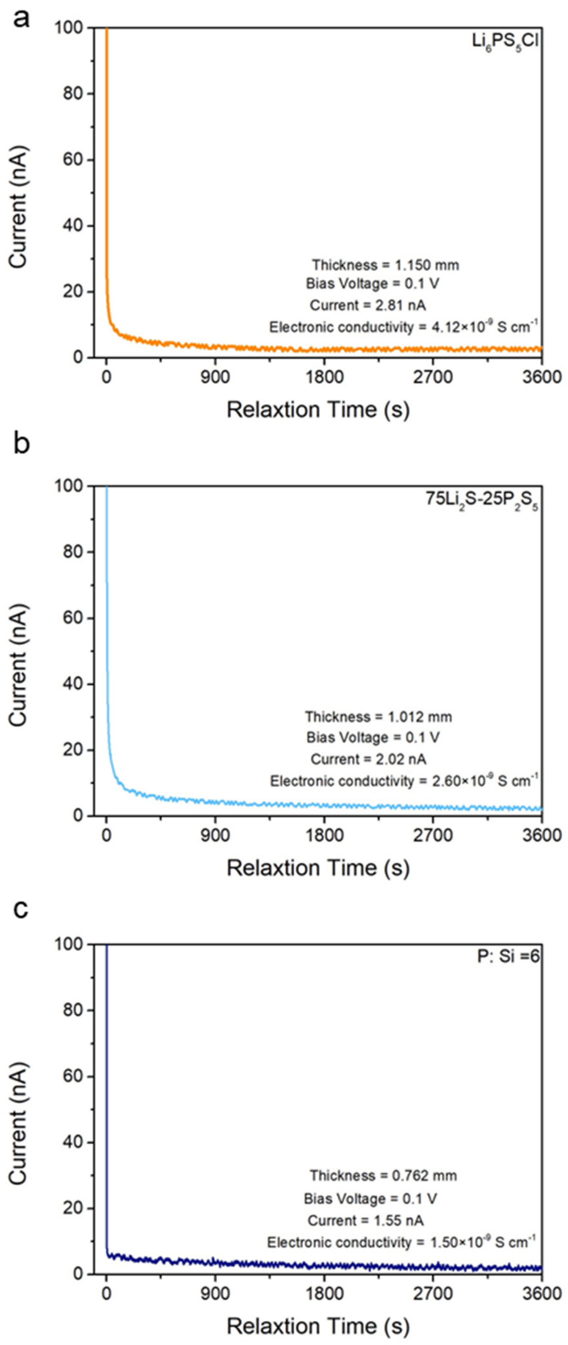

Fig. S23 The measurement of electronic conductivity for state-of-the-art sulfides and the P:Si = 6 electrolyte. **a-c** The DC polarization curves of (**a**) Li_6PS_5Cl, (**b**) $75Li_2S$-$25P_2S_5$ and (**c**) P:Si = 6 electrolyte at a bias voltage of 0.1V.

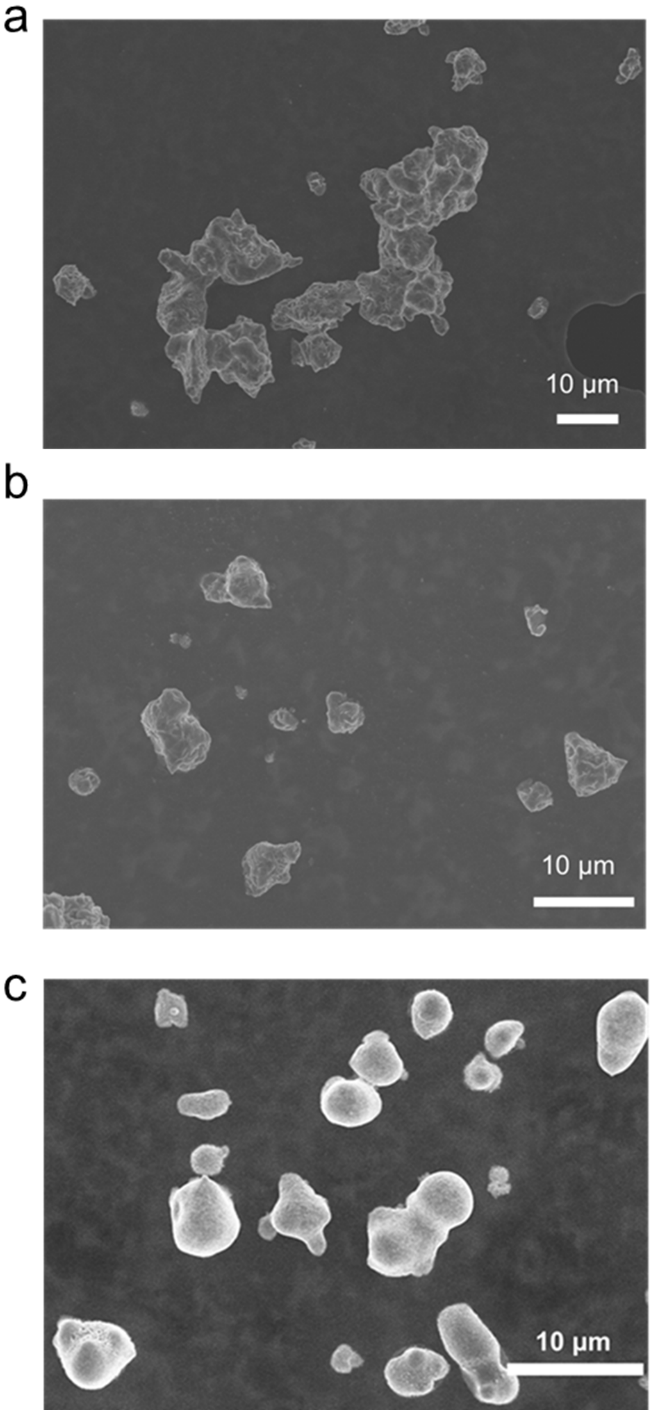

Fig. S24 The morphology of electrolyte particles for state-of-the-art sulfides and the P:Si = 6 electrolyte. **a** The morphology of distributing Li_6PS_5Cl particles. **b** The morphology of distributing $75Li_2S$-$25P_2S_5$ particles. **c** The morphology of distributing P:Si = 6 particles.

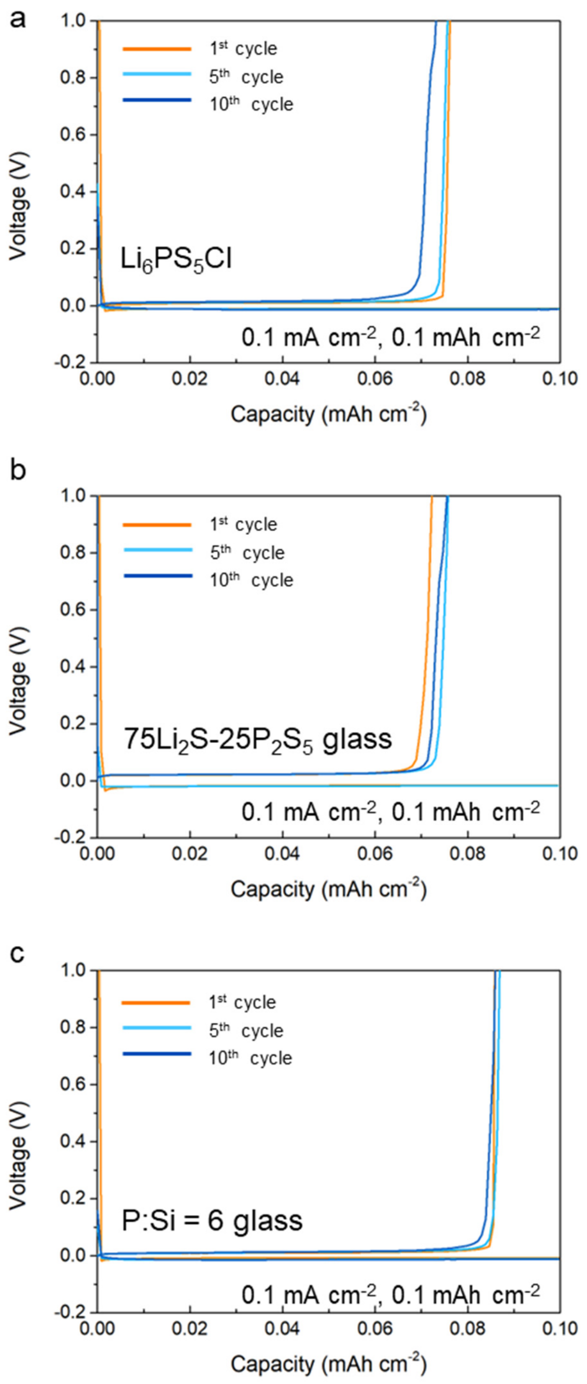

Fig. S25 The voltage profiles of Li||SS half cells**. a-c** The voltage profiles of Li||SS half cells using (**a**) Li_6PS_5Cl, (**b**) $75Li_2S$-$25P_2S_5$ and (**c**) P:Si = 6 electrolytes as the interlayer in the 1st, 5th and 10th cycles.

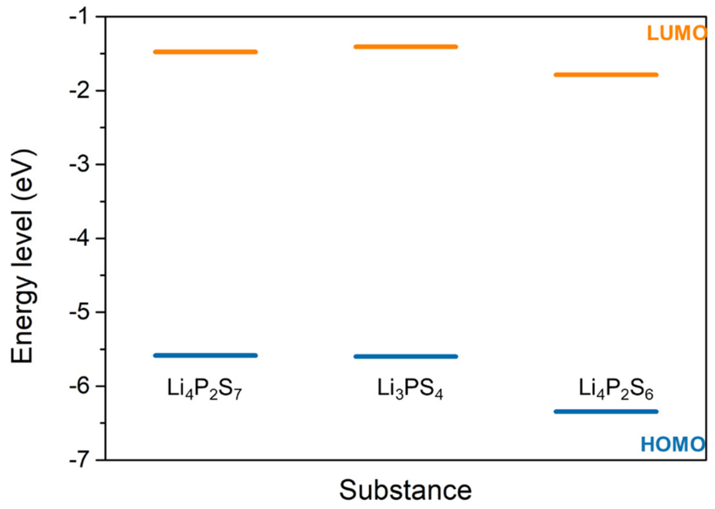

Fig. S26 The calculated HOMO/LUMO values for $Li_4P_2S_7$, Li_3PS_4 and $Li_4P_2S_6$ molecules.

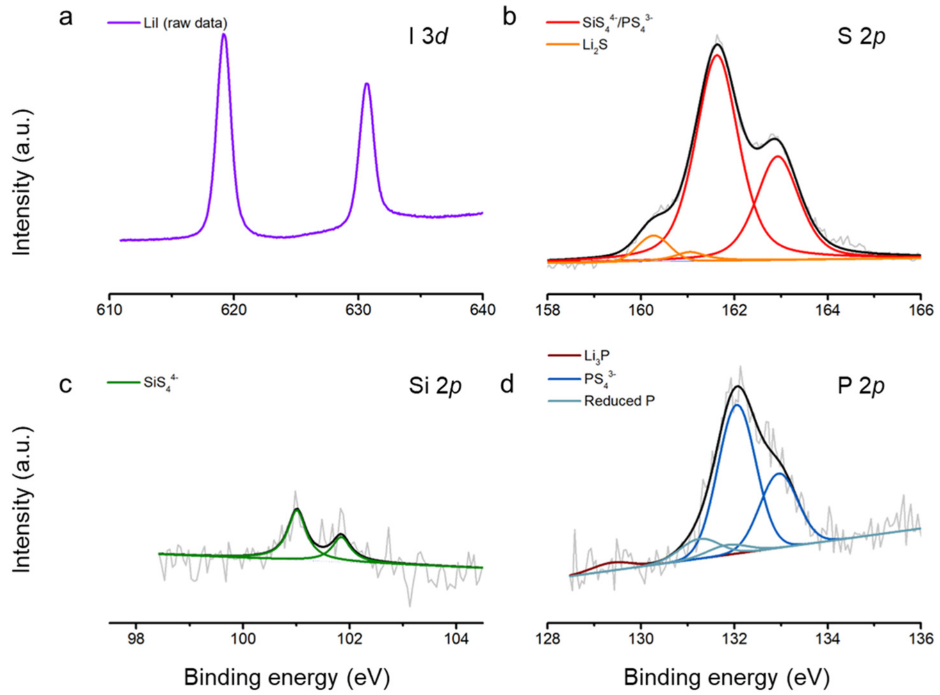

Fig. S27 The XPS characterization of the Li/P:Si = 6 interface. **a** The I 3*d* spectrum of the Li/P:Si = 6 interface. **b** The S 2*p* spectrum of the Li/P:Si = 6 interface. The raw data is plotted in a grey line. **c** The Si 2*p* spectrum of the Li/P:Si = 6 interface. The raw data is plotted in a grey line. **d** The P 2*p* spectrum of the Li/P:Si = 6 interface. The raw data is plotted in a grey line.

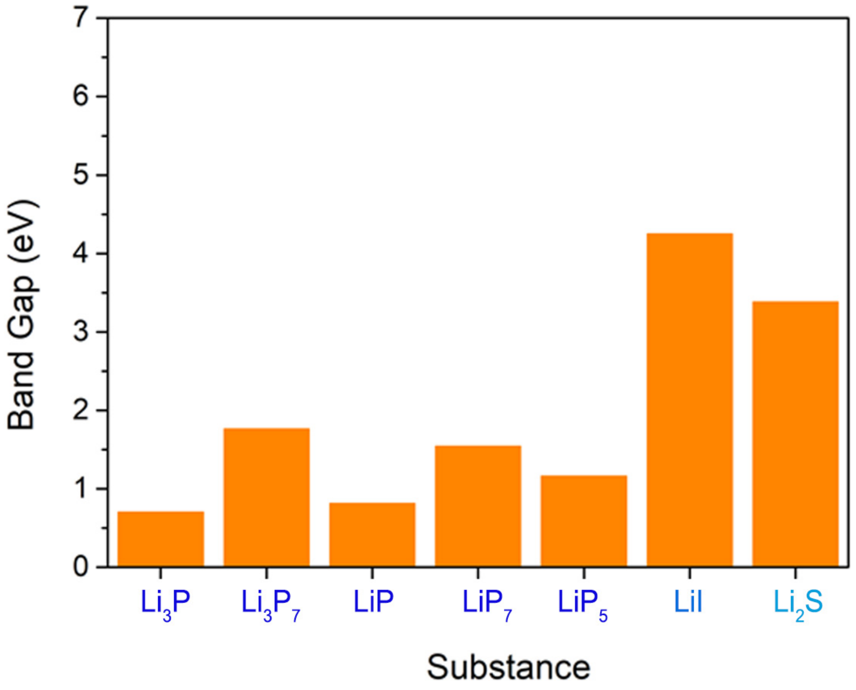

Fig. S28 The summarized band gap of the possible SEI components in L-P-S-I systems from Materials Project.

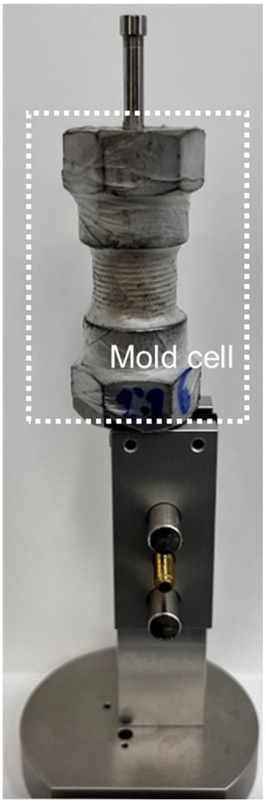

Fig. S29 The optical image of the mold cell and the holder for X-ray CT tests.

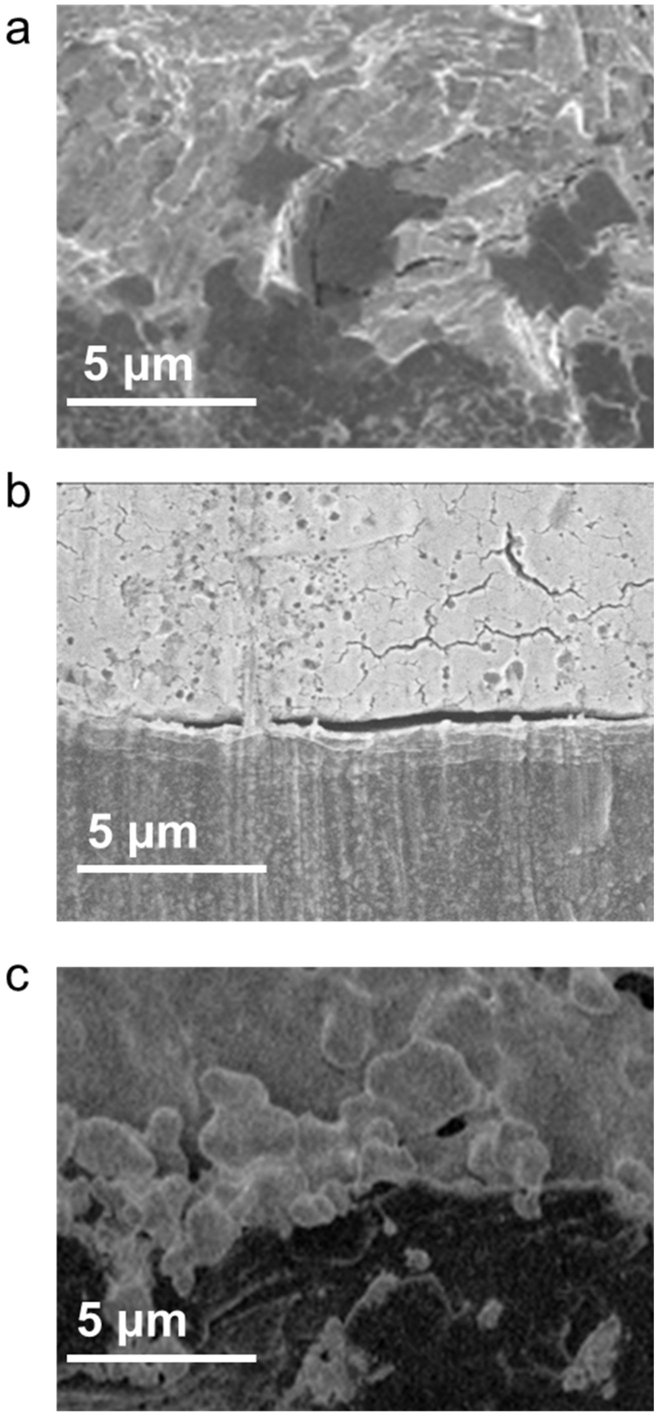

Fig. S30 The morphology of Li/SSE interface after cycling. **a** The cross-sectional image of Li/Li_6PS_5Cl interface after cycles. **b** The cross-sectional image of Li/75Li_2S-25P_2S_5 interface after cycles. **c** The cross-sectional image of Li/P:Si = 6 interface after cycles.

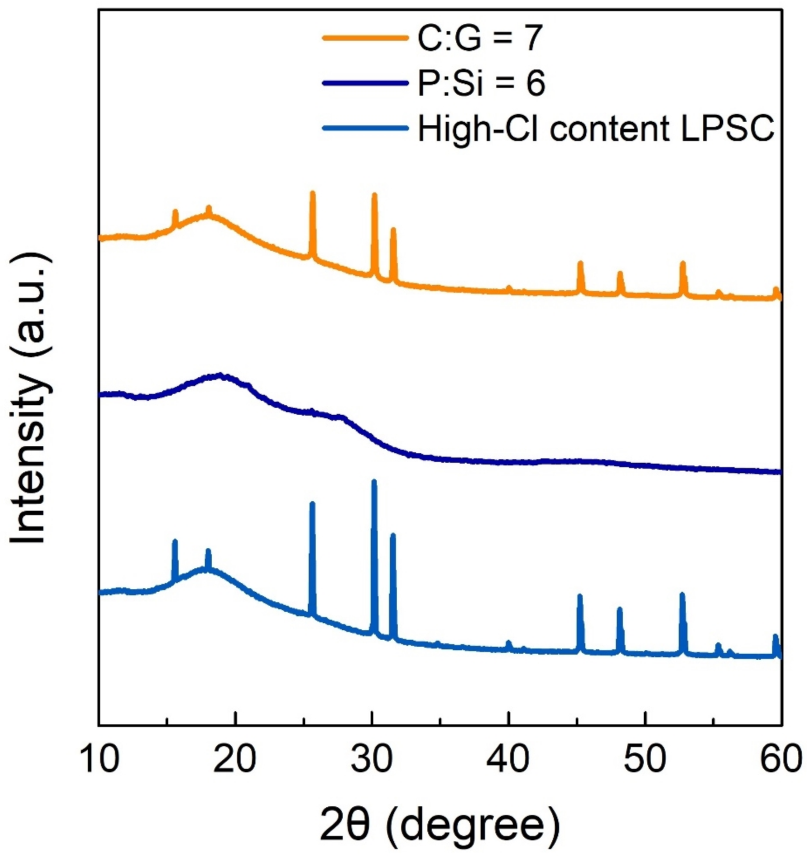

Fig. S31 XRD spectra of C:G = 7, P:Si = 6 and high-Cl content LPSC electrolytes.

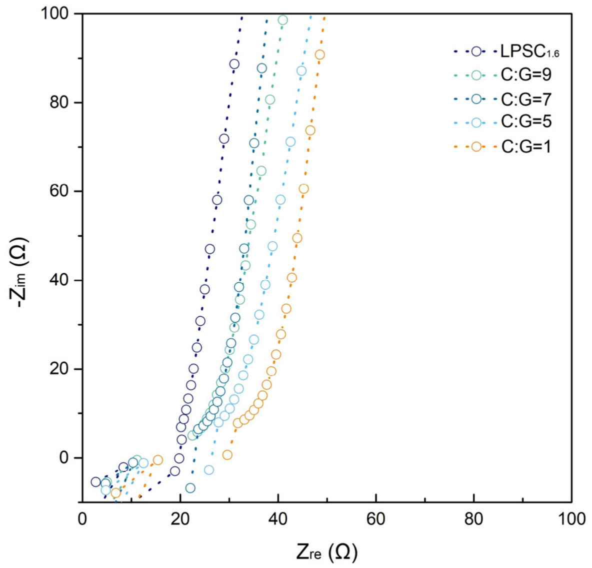

Fig. S32 Nyquist plots of electrolytes with different C/G ratios at 298 K.

Table S7 The summary of the relative density of composite electrolytes with different C/G ratios.

Composition	Theoretical Density / (g cm^{-3})	Weight / (mg)	Thickness / (mm)	Real Density / (g cm^{-3})	Relative Density
$LPSCl_{1.6}$	1.880	139.0	1.122	1.578	0.839
C:G = 9	1.928	142.3	1.079	1.681	0.872
C:G = 7	1.940	145.0	1.068	1.730	0.892
C:G = 5	1.961	149.3	1.084	1.754	0.894
C:G = 1	2.146	147.5	0.971	1.935	0.902

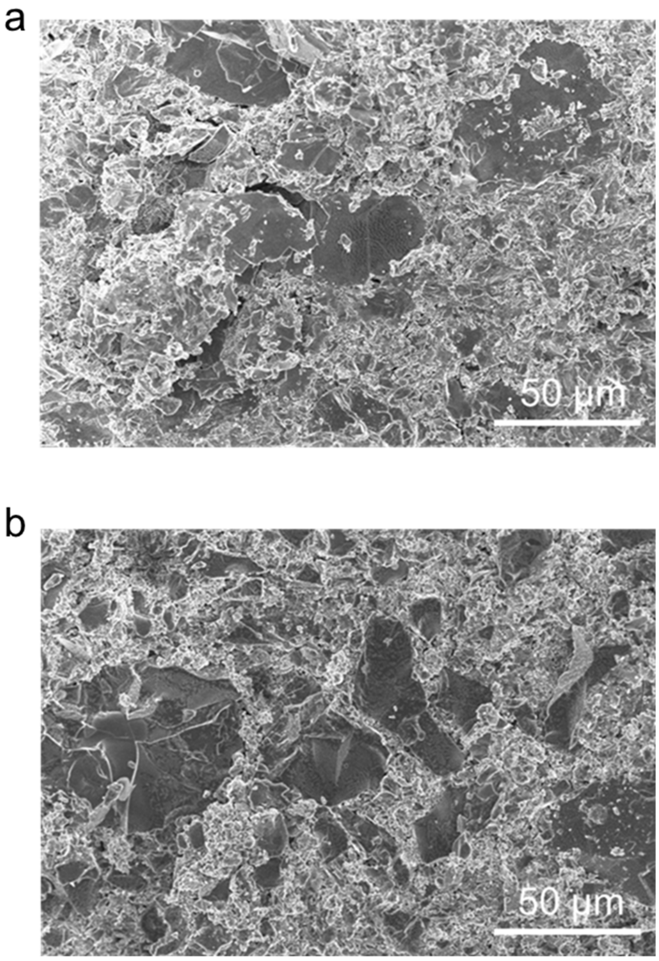

Fig. S33 The morphology comparison between cold-pressed $Li_{5.4}PS_{4.4}Cl_{1.6}$ and C:G = 7. **a** The surface morphology of the cold-pressed $Li_{5.4}PS_{4.4}Cl_{1.6}$ electrolyte. **b** The surface morphology of the C:G = 7 electrolyte.

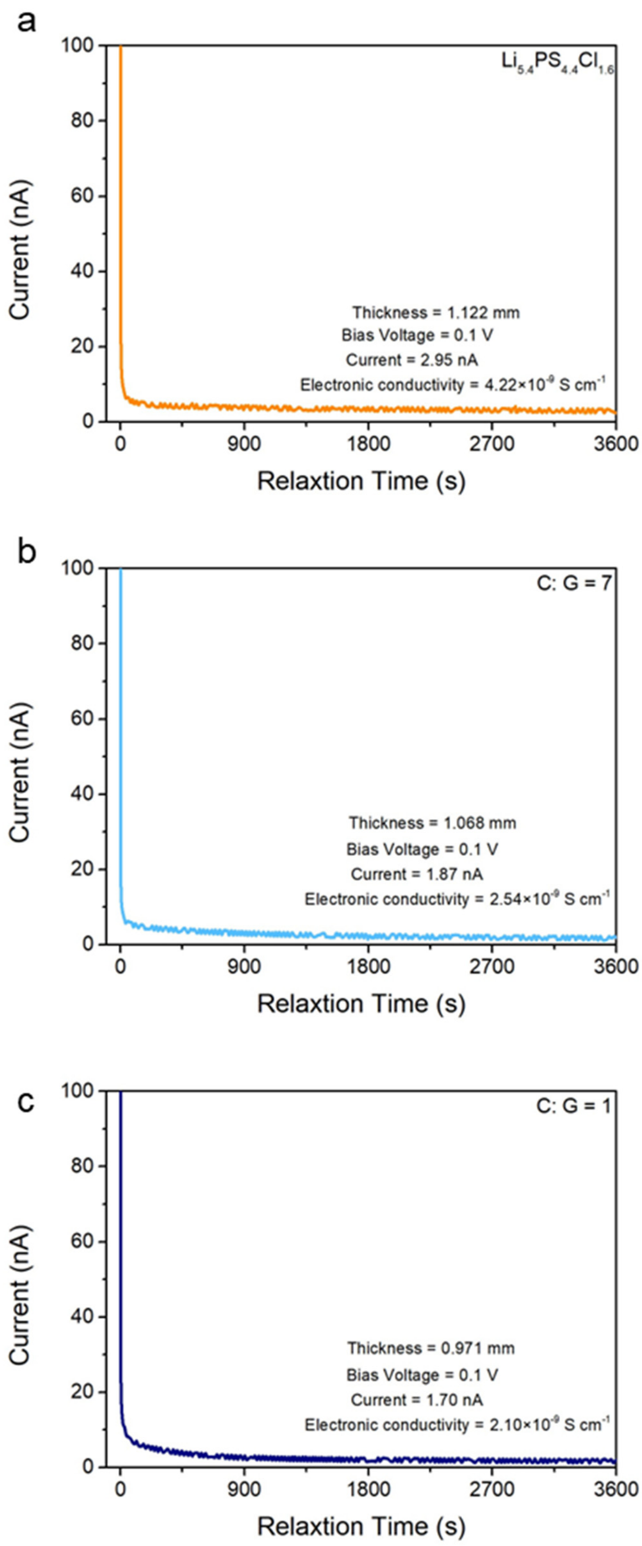

Fig. S34 The measurement of electronic conductivity for $Li_{5.4}PS_{4.4}Cl_{1.6}$, C:G = 7 and C:G = 1 electrolytes. **a-c** The DC polarization curves of (**a**) $Li_{5.4}PS_{4.4}Cl_{1.6}$, (**b**) C:G = 7, and (**c**) C:G = 1 electrolyte at a bias voltage of 0.1V.

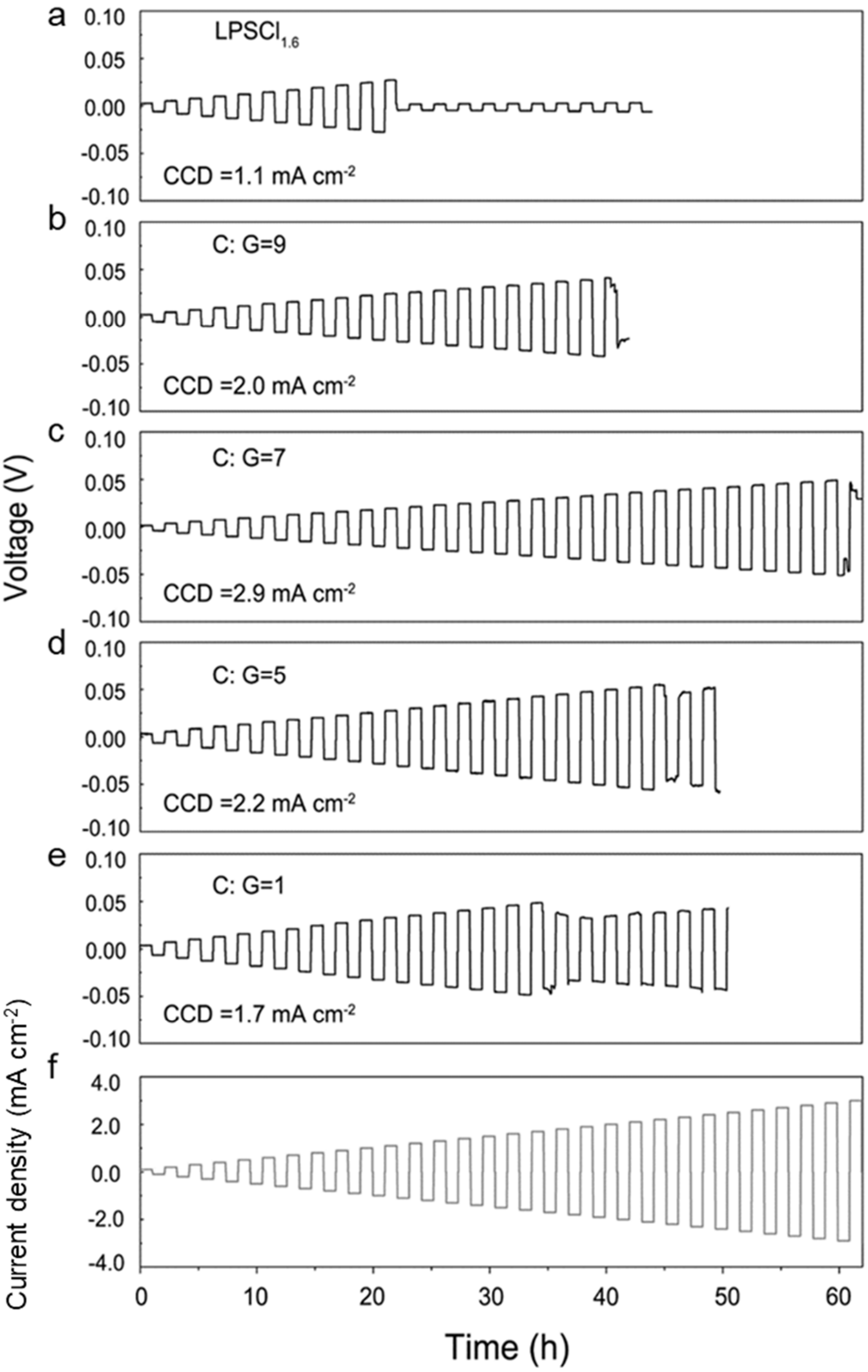

Fig. S35 The CCD tests of the glassy/crystalline composite electrolytes**. a-e** The voltage profiles of $Li_{5.4}PS_{4.4}Cl_{1.6}$ (**a**), C:G = 9 (**b**), C:G = 7 (**c**), C:G = 5 (**d**) and C:G = 1 (**e**) electrolytes during the CCD test. **f** The current density profile of the CCD test. The step size of current density was set to be 0.1 mA cm^{-2}. And the charging/discharing time was set to be 1 h.

Table S8 The summarization of the reported CCD values for sulfide-based SEs at 298 K. (discharging/charging time = 1 h, bare Li as the electrode).

Sulfide-based SE	CCD / (mA cm^{-2})	Capacity / (mAh cm^{-2})	Ref.
C : G = 7	2.90	2.90	*This Work
$LI_{5.5}PS_{4.5}Cl_{1.5}$	1.40	1.40	14
Li_6PS_5Cl (pressure hold for 30 min)	2.15	2.15	15
$Li_{6.25}PS_{4.75}ClN_{0.25}$	1.52	1.52	16
$Li_7P_{2.88}Nb_{0.12}S_{10.7}O_{0.3}$	1.16	1.16	17
$Li_6PS_{4.7}O_{0.3}Br$	0.89	0.89	18
Li_6PS_5Cl	0.60	0.60	*This Work
$LI_{5.4}PS_{4.4}Cl_{1.6}$	1.10	1.10	* This Work
$Li_7P_3S_{11}$	0.76	0.76	19
$Li_7P_{2.9}Si_{0.05}S_{10.85}$	0.80	0.80	19

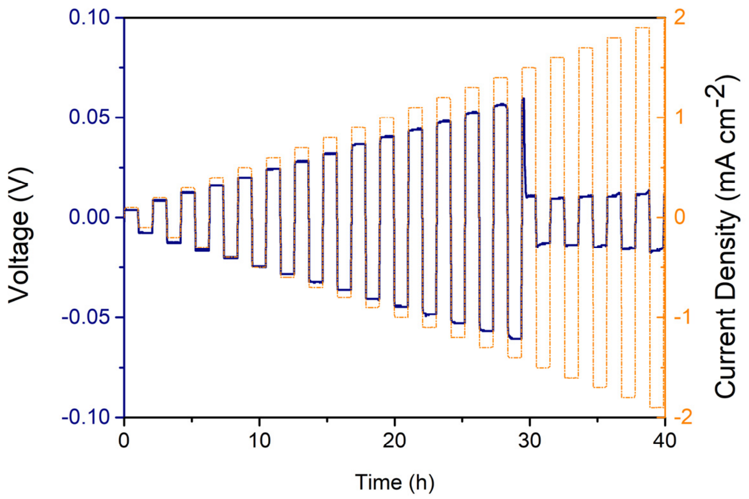

Fig. S36 The CCD profile of the $Li_{5.4}PS_{4.4}Cl_{1.6}$/$75Li_2S$-$25P_2S_5$ composite electrolyte, with a weight ratio of $75Li_2S$-$25P_2S_5$ = 12.5 %.

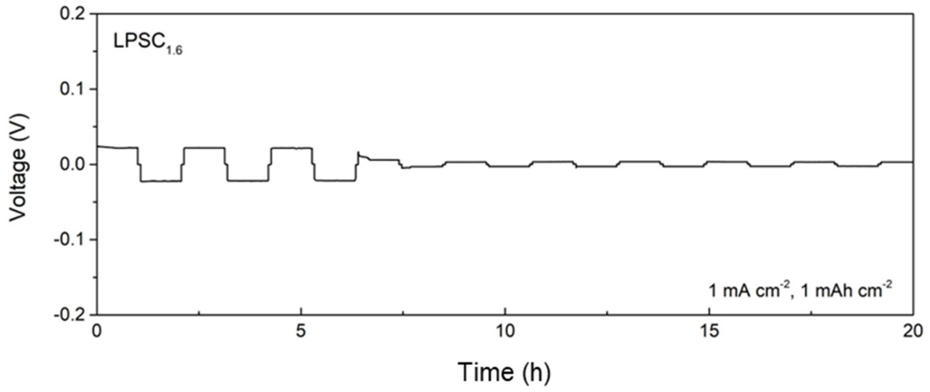

Fig. S37 The galvanostatic discharging/charging profiles of the Li symmetric cell using the $Li_{5.4}PS_{4.4}Cl_{1.6}$ electrolyte at a current density of 1 mA cm^{-2} and a cut-off capacity of 1 mAh cm^{-2}.

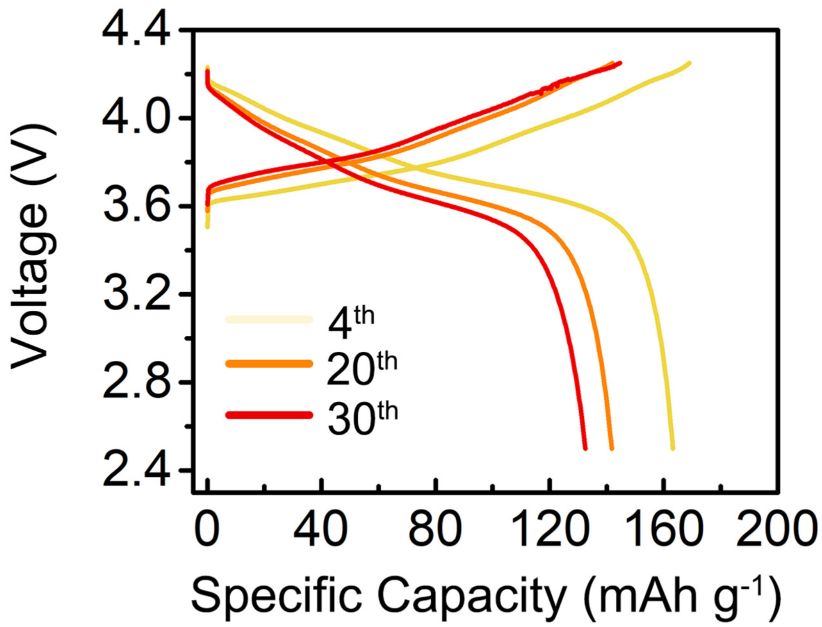

Fig. S38 The voltage profiles of Li| $Li_{5.4}PS_{4.4}Cl_{1.6}$ |NCM83125 full cell at different cycles.

Table S9 The summarization of the reported Li symmetric cells' (using bare Li metal) performances for sulfide-based SEs at 298 K.

Sulfide-based SE	Current Density / (mA cm^{-2})	Capacity / (mAh cm^{-2})	Cycling Time / (h)	Ref.
C : G = 7	1.0	3.0	900	*This Work
C : G = 7	1.0	1.0	1200	*This Work
P : Si = 6	0.1	0.5	8000	*This Work
P : Si = 6	0.2	0.2	2000	*This Work
P : Si = 6	1.0	0.1	1500	*This Work
$Li_{6.25}PS_{4.75}ClN_{0.25}$	1.0	1.0	200	16
$Li_{6.25}PS_{4.75}ClN_{0.25}$	0.5	0.5	1000	16
$Li_7P_{2.88}Nb_{0.12}S_{10.7}O_{0.3}$	0.2	0.2	300	17
$LPSI_{1.4}$-gc	0.2	0.2	3200	20
$Li_6PS_{4.7}O_{0.3}Br$	0.4	0.4	350	18
$LiF@Li_{10}GeP_2S_{12}$	0.1	0.1	1000	21
PEGME-SPE/LPSC	1.0	0.5	1000	22
LPSI-20Sn	1.26	1.0	200	23
$Li_{5.5}PS_{4.425}O_{0.075}Cl_{1.5}$	0.4	0.2	150	24
$Li_{6.988}P_{2.994}Nb_{0.2}S_{10.934}O_{0.6}$	0.1	0.1	100	25
$Li_{5.6}Cu_{0.2}PS_{4.8}Br_{1.2}$	0.2	0.2	1200	26
$Li_{5.5}PS_{4.5}Cl_{1.5}$	0.2	0.2	500	14
$Li_{5.5}PS_{4.5}Cl_{1.5}$	0.5	0.5	200	14
$Li_{6.04}P_{0.98}Bi_{0.02}S_{4.97}O_{0.03}Cl$	0.1	0.1	600	27

Table S10 The summarization of the reported high-voltage ASSLMBs' performances at 298 K.

Ref.	Initial Discharging Capacity / (mAh g^{-1})	Total cycles / (n)	Cathode	Interlayer	Capacity retention / (%)
28	125	75	NCM111	$Li_{10}SiP_2S_{12}$	91
29	131	500	$LiCoO_2$	$Li_{10}GeP_2S_{12}$	87
30	121	25	$LiCoO_2$	@$Li_{3.15}Ge_{0.15}P_{0.85}S_4$/ 77.5$Li_2S-22.5P_2S_5$	90
31	134	50	$LiCoO_2$	@$Li_{10}GeP_2S_{12}$/ $Li_3P_{0.98}Sb_{0.02}S_{3.95}O_{0.05}$	79
32	110	100	NCA	75Li_2S-25P_2S_5	84
33	170	40	NCM622	0.5LiI−Li_3PS_4	74
16	136	100	$LiCoO_2$	$Li_{6.25}PS_{4.75}ClN_{0.25}$	87
34	133	80	$LiCoO_2$	$Li_{5.4}PS_{4.4}Cl_{1.6}$	71
14	151	100	NCM811	$Li_{5.5}PS_{4.5}Cl_{1.5}$	80
35	113	100	$LiCoO_2$	Li_6PS_5Cl-MgF_2	92
36	163	100	NCM811	Li_6PS_5Cl	90
*This work	172	500	NCM83125	C:G =7	82

@ Two layers of electrolytes were applied in the ASSLMBs.

Reference

1. Ohtomo T, Hayashi A, Tatsumisago M, Tsuchida Y, Hama S, Kawamoto K. All-solid-state lithium secondary batteries using the $75Li_2S{\cdot}25P_2S_5$ glass and the $70Li_2S{\cdot}30P_2S_5$ glass–ceramic as solid electrolytes. *J Power Sources* **233**, 231-235 (2013).

2. Han FD, Yue J, Zhu XY, Wang CS. Suppressing Li dendrite formation in Li_2S-P_2S_5 solid electrolyte by LiI incorporation. *Adv Energy Mater* **8**, 1703644 (2018).

3. Kaup K*, et al.* A lithium oxythioborosilicate solid electrolyte glass with superionic conductivity. *Adv Energy Mater* **10**, 1902783 (2020).

4. Yamauchi A, Sakuda A, Hayashi A, Tatsumisago M. Preparation and ionic conductivities of $(100-x)(0.75Li_2S{\cdot}0.25P_2S_5){\cdot}xLiBH_4$ glass electrolytes. *J Power Sources* **244**, 707-710 (2013).

5. Wada H, Menetrier M, Levasseur A, Hagenmuller P. Preparation and ionic conductivity of new B_2S_3-Li_2S-LiI glasses. *Mat Res Bull,* **18**, 189-193 (1983).

6. Kennedy JH, Zhang ZM, Eckert H. Ionically conductive suldie-based lithium conductors. *J Non-Crystalline Solids* **123**, 328-338 (1990).

7. Cengiz M, Oh H, Lee SH. Lithium dendrite growth suppression and ionic conductivity of Li_2S-P_2S_5-P_2O_5 glass solid electrolytes prepared by mechanical milling. *J Electrochem Soc* **166**, A3997-4004 (2019).

8. Ohtomo T, Hayashi A, Tatsumisago M, Kawamoto K. Glass electrolytes with high ion conductivity and high chemical stability in the system LiI-Li_2O-Li_2S-P_2S_5. *Electrochem* **81**, 428-431 (2013).

9. Wang YQ, Matsuyama T, Deguchi M, Hayashi A, Nakao A, Tatsumisago M. X-ray photoelectron spectroscopy for sulfide glass electrolytes in the systems Li_2S-P_2S_5 and Li_2S-P_2S_5-LiBr. *J Ceram Soc JPN* **124**, 597-601 (2016).

10. Hayashi A, Muramatsu H, Ohtomo T, Hama S, Tatsumisago M. Improved chemical stability and cyclability in Li_2S–P_2S_5–P_2O_5–ZnO composite electrolytes for all-solid-state rechargeable lithium batteries. *J Alloy Compd* **591**, 247-250 (2014).

11. Zhao R*, et al.* New amorphous oxy-sulfide solid electrolyte material: anion exchange, electrochemical properties, and lithium dendrite suppression via in situ interfacial modification. *ACS Appl Mater Interfaces* **13**, 26841-26852 (2021).

12. Yersak TA, Kang CS, Salvador JR, Nicholas PWP, Cai M. Sulfide glass solid-state electrolyte separators for Li metal batteries: using an interlayer to increase rate

performance and reduce stack pressure. *Mat Adv* **3**, 3562 (2022).

13. Kato A, Yamamoto M, Sakuda A, Hayashi A, Tatsumisago M. Mechanical properties of $Li_2S–P_2S_5$ glasses with lithium halides and application in all-solid-state batteries. *ACS Appl Energy Mater* **1**, 1002-1007 (2018).

14. Liu Y*, et al.* Revealing the Impact of Cl substitution on the crystallization behavior and interfacial stability of superionic lithium argyrodites. *Adv Func Mater* **32**, 2207978 (2022).

15. Ham SY*, et al.* Assessing the critical current density of all-solid-state Li metal symmetric and full cells. *Energy Storage Mater* **55**, 455-462 (2023).

16. Liu Y*, et al.* In situ formation of a Li_3N-rich interface between lithium and argyrodite solid electrolyte enabled by nitrogen doping. *J Mater Chem A* **9**, 13531-13539 (2021).

17. Jiang Z*, et al.* Improved ionic conductivity and Li dendrite suppression capability toward $Li_7P_3S_{11}$-Based Solid electrolytes triggered by Nb and O cosubstitution. *ACS Appl Mater Interfaces* **12**, 54662-54670 (2020).

18. Zhang ZX*, et al.* All-in-one improvement toward Li_6PS_5Br-based solid electrolytes triggered by compositional tune. *J Power Sources* **410-411**, 162-170 (2019).

19. Wang ZX*, et al.* Doping effects of metal cation on sulfide solid electrolyte/lithium metal interface. *Nano Energy* **84**, 105906 (2021).

20. Liu Y*, et al.* Ultrafast synthesis of I-rich lithium argyrodite glass-ceramic electrolyte with high ionic conductivity. *Adv Mater* **34**, 2107346 (2022).

21. Jin YM*, et al.* Fluorinated $Li_{10}GeP_2S_{12}$ enables stable all-solid-state lithium batteries. *Adv Mater*, **35**, 2211047 (2023).

22. Yang XF*, et al.* Grain boundary electronic insulation for high-performance all-solid-state lithium batteries. *Angew Chem Int Ed* **62**, 202215680 (2023).

23. Zhao FP*, et al.* A versatile Sn‐substituted argyrodite sulfide electrolyte for all‐solid‐state Li metal batteries. *Adv Energy Mater* **10**, 1903422 (2020).

24. Peng LF*, et al.* Enhancing moisture and electrochemical stability of the $Li_{5.5}PS_{4.5}Cl_{1.5}$ electrolyte by oxygen doping. *ACS Appl Mater Interfaces* **14**, 4179-4185 (2022).

25. Ahmad N*, et al.* Enhanced air stability and high Li-ion conductivity of $Li_{6.988}P_{2.994}Nb_{0.2}S_{10.934}O_{0.6}$ glass-ceramic electrolyte for all-solid-state lithium-sulfur batteries. *ACS Appl Mater Interfaces* **12**, 21548-21558 (2020).

26. Jiang Z*, et al.* Enhanced air stability and interfacial compatibility of Li-argyrodite sulfide electrolyte triggered by CuBr Co-substitution for all-solid-state lithium batteries. *Energy Storage Mater* **56**, 300-309 (2023).

27. Liu H*, et al.* High air stability and excellent Li metal compatibility of argyrodite‐based electrolyte enabling superior all‐solid‐state Li metal batteries. *Adv Func Mater* **32**, 2203858 (2022).

28. Whiteley JM, Woo JH, Hu EY, Nam KW, Lee SH. Empowering the lithium metal battery through a silicon-based superionic conductor. *J Electrochem Soc* **161**, A1812-1817 (2014).

29. Zhang ZH*, et al.* Interface re-engineering of $Li_{10}GeP_2S_{12}$ electrolyte and lithium anode for all-solid-atate lithium batteries with ultralong cycle life. *ACS Appl Mater Interfaces* **10**, 2556-2565 (2018).

30. Woo JH*, et al.* Nanoscale interface modification of $LiCoO_2$ by Al_2O_3 atomic layer deposition for solid-state Li batteries. *J Electrochem Soc* **159**, A1120-1124 (2012).

31. Xie DJ*, et al.* High ion conductive Sb_2O_5-doped β-Li_3PS_4 with excellent stability against Li for all-solid-state Lithium batteries. *J Power Sources* **389**, 140-147 (2018).

32. Ulissi U, Agostini M, Ito S, Aihara Y, Hassoun J. All solid-state battery using layered oxide cathode, lithium-carbon composite anode and thio-LISICON electrolyte. *Solid State Ionics* **296**, 13-17 (2016).

33. Choi SJ*, et al.* LiI-doped sulfide solid electrolyte: enabling a high-capacity slurry-cast electrode by low-temperature post-sintering for practical all-solid-state lithium batteries. *ACS Appl Mater Interfaces* **10**, 31404-31412 (2018).

34. Su H*, et al.* Stabilizing the interphase between Li and argyrodite electrolyte through synergistic phosphating process for all-solid-state lithium batteries. *Nano Energy* **96**, 107104 (2022).

35. Liu C*, et al.* Electron redistribution enables redox-resistible Li_6PS_5Cl towards high-performance all-solid-state lithium batteries. *Angew Chem Int Ed*, **62**, 202302655 (2023).

36. Kim J*, et al.* High‐performance all‐solid‐state batteries enabled by intimate interfacial contact between the cathode and sulfide‐based solid electrolytes. *Adv Func Mater* **33**, 2211355 (2023).
