# Supplementary material for: Deciphering the critical role of interstitial volume in glassy sulfide superionic conductors
Source: Nat Commun. 2024 Mar 22;15:2552. doi: 10.1038/s41467-024-46798-4 (PMC10957893; doi:10.1038/s41467-024-46798-4)
Supplement: Supplementary file 3 — Description of Additional Supplementary Files [file 41467_2024_46798_MOESM3_ESM.pdf]

Supplementary Data 1. The structure data of Si-substituted Li_4PS_4I and Li_4PS_4I before and after AIMD simulations.
